# Notes on a Month of Influenza at a Base Hospital in France

**Published:** 1919

**Authors:** 


### ABSTRACTS

### *Notes on a Month of Influenza at a Base Hospital in France.*

By Captain S. Bradbury and Captain E. B. Krumbhaar.

The authors review results secured in five hundred and thirty-three cases of influenza which represented the pinnacle of the epidemic at their hospital. Of these cases some were already convalescent and others had developed pneumonia before admission.

The onset was sudden, causing severe prostration, headache behind the eyes, and often a chilly sensation. Constipation was usually present, though some cases had diarrhea with vomiting. Pulse was slow and respiration normal. Severe cough, cyanosis, and pain in upper abdomen. Relapse frequent but usually mild.

Pneumonia began either during fever or two or three days after its cessation. It was indicated by elevated temperature, cyanosis, quick pulse and respiration, and cough with purulent sputum. A few patients had small frothy hemorrhages before death. At onset there was suppression or breath sounds at one or both bases with crepitant rales. Where death occurred early in the disease, cyanosis rapidly increased with accompanying delirium, and in later cases the cyanosis continued, the patient wasted rapidly, cough was severe, delirium almost constant, and, in one or two weeks, the patient collapsed breaking into a drenching sweat, and no stimulation was of any effect. Sometimes, while doing well, the patient complained of pain in one lung and died in a few hours.

There were many complications covering jaundice, urticarial rash, septic rash, emboli of the fingers or thigh — in nearly every case fatal.

Where death did not intervene the temperature fell slowly to normal, though the patient remained ill from two to five days afterward. Convalescence was tedious.

In twenty-eight autopsies, pneumonia was present in every one and eleven showed streptococcic abscesses of the lung. Two had pyemia with abscesses of liver, spleen, and kidneys. In eight the lung abscesses were associated with streptococcic pleurisy, and three others had sub-pleural abscesses ready to burst. Of those lungs not complicated by abscesses the majority were of a new type — portions of the lobe were homogeneous, firm, heavy, wet, and red, with a peculiar boggy appearance. Some of them floated low in water and others sank. The scraped fluid gave the impression of a revolving lobar pneumonia. In the older cases where the homogeneity of the lungs was less marked the tissue was found divided by moist, raised, gray, granulated belts. Alveoli were not prominent and many were packed with red blood cells fibrin, serous fluid and nucleated cells. Small round cells and plasma usually predominated, though sections from paler and granular areas showed more polymorphonuclears. The time element bore little relation to the incidence of the disease.

Smears and cultures were made in eighteen cases postmortem, and in all streptococcus hemolyticus were found together with pneumococci. The abscesses and septic pleural fluid invariably contained a majority of streptococcus hemolyticus. There were also pneumococci which were responsible for the consolidation. If B. Pfeiffer had been present they had disappeared before death.

A tardy effort to protect by means of vaccines was reported, and the conclusion reached that " a favorable influence was exerted on the course of the influenza ".

Leucocyte counts were made on twenty patients, ten of whom had pneumonia, and leucocytosis was present in six, all of whom had pneumonia; a leukopenia occurred in seven cases, of whom five had pneumonia. The chief changes were apparently due to variation in the polymorphonuclears. Three of the seven leukopenic cases died and four with leucocytosis, nevertheless the authors consider leucocytosis a good omen in the presence of influenza complications.

Urine in all the mild and many of the severe cases was normal; other severe cases showed a cloud of albumin and hyaline and granular casts.

*Summary.*

1. 83.3 o/o of cases were uncomplicated. 16.7 o/o developed pneumonia, of whom fifty per cent. died.

2. Uncomplicated cases were typical.

Frequency and high mortality of pulmonary complications was striking, also the tendency to hemorrhage from lungs, nose and beneath the skin.

3. In twenty-eight autopsies all showed pleurisy and pneumonia. Eleven exhibited multiple streptococcic abscesses and eight of them septic pleurisy.

4. Pneumococci predominated in the earlier consolidations and hemolytic streptococci in the abscesses.

5. Polyvalent vaccines were given in thirty-three febrile cases and none developed pneumonia.

